# Adiposis Dolorosa

**Published:** 1899-12-23

**Authors:** 


### ADIPOSIS DOLOROSA.

Dr. Hale White[1] records a case of adiposis dolorosa to which we would direct attention, because it is an interesting example of a disease which in its fully-developed condition is of some rarity, The points to look out for in such cases are the lumpy deposits of fat, and the pains and tenderness on pressure. So far all the observed cases have been in women. The onset is usually noticed between the ages of 40 and 60. The first thing noticed is that fatty masses appear on the trunk or limbs, sometimes symmetrically arranged. At the same time the patient becomes generally obese, sometimes enormously so, but even in these cases here and there large lumps are to be found. Pain is a striking symptom, and is usually felt as soon as, or even before, the fatty masses. It also may be felt in parts which do not become fat. A sense of coldness is often complained of, but when the case is well developed there is generally diminished power of appreciating pain, touch, heat, and cold. The masses of fat are often intensely tender when grasped. Both muscular and mental power become enfeebled. A short account of this disease may be found in Allbutt's "System of Medicine," vol. vi., in which it is said: "The disorder differs from other forms of obesity in its partial and lumpy distribution, in the presence of pains, and sometimes of impaired sensibility; and from myxœdema in the freedom of face, hands, and feet, and in the absence of the pronounced mental and trophic manifestations of this disease." Hale White seems to lay more stress on the mental condition. Thyroid extract was tried, but without benefit.

[1] Brit. Med. Jour., Dec. 2.

